# Hepatitis B Virus X Protein Upregulates mTOR Signaling through IKKβ to Increase Cell Proliferation and VEGF Production in Hepatocellular Carcinoma

**DOI:** 10.1371/journal.pone.0041931

**Published:** 2012-07-27

**Authors:** Chia-Jui Yen, Yih-Jyh Lin, Chia-Sheng Yen, Hung-Wen Tsai, Ting-Fen Tsai, Kwang-Yu Chang, Wei-Chien Huang, Pin-Wen Lin, Chi-Wu Chiang, Ting-Tsung Chang

**Affiliations:** 1 Institute of Clinical Medicine, National Cheng Kung University College of Medicine and Hospital, Tainan, Taiwan; 2 Department of Internal Medicine, National Cheng Kung University College of Medicine and Hospital, Tainan, Taiwan; 3 Department of Surgery, National Cheng Kung University College of Medicine and Hospital, Tainan, Taiwan; 4 Department of General Surgery, Chi-Mei Medical Center, Tainan, Taiwan; 5 Department of Pathology, National Cheng Kung University College of Medicine and Hospital, Tainan, Taiwan; 6 Department of Life Sciences and Institute of Genome Sciences, National Yang-Ming University, Taipei, Taiwan; 7 Institute of Cancer Research, National Health Research Institutes, Tainan, Taiwan; 8 Center for Molecular Medicine and Graduate Institute of Cancer Biology, China Medical University and Hospital, Taichung, Taiwan; 9 Institute of Molecular Medicine, College of Medicine, National Cheng Kung University, Tainan, Taiwan; Drexel University College of Medicine, United States of America

## Abstract

Hepatocellular carcinoma (HCC), a major cause of cancer-related death in Southeast Asia, is frequently associated with hepatitis B virus (HBV) infection. HBV X protein (HBx), encoded by a viral non-structural gene, is a multifunctional regulator in HBV-associated tumor development. We investigated novel signaling pathways underlying HBx-induced liver tumorigenesis and found that the signaling pathway involving IκB kinase β (IKKβ), tuberous sclerosis complex 1 (TSC1), and mammalian target of rapamycin (mTOR) downstream effector S6 kinase (S6K1), was upregulated when HBx was overexpressed in hepatoma cells. HBx-induced S6K1 activation was reversed by IKKβ inhibitor Bay 11-7082 or silencing IKKβ expression using siRNA. HBx upregulated cell proliferation and vascular endothelial growth factor (VEGF) production, and these HBx-upregulated phenotypes were abolished by treatment with IKKβ inhibitor Bay 11-7082 or mTOR inhibitor rapamycin. The association of HBx-modulated IKKβ/mTOR/S6K1 signaling with liver tumorigenesis was verified in a HBx transgenic mouse model in which pIKKβ, pS6K1, and VEGF expression was found to be higher in cancerous than non-cancerous liver tissues. Furthermore, we also found that pIKKβ levels were strongly correlated with pTSC1 and pS6K1 levels in HBV-associated hepatoma tissue specimens taken from 95 patients, and that higher pIKKβ, pTSC1, and pS6K1 levels were correlated with a poor prognosis in these patients. Taken together, our findings demonstrate that HBx deregulates TSC1/mTOR signaling through IKKβ, which is crucially linked to HBV-associated HCC development.

## Introduction

Hepatocellular carcinoma (HCC), which occurs frequently in Southeast Asia, is one of the most important causes of cancer-related death in the world [1], [2], [3]. According to epidemiological studies [4], [5], [6], [7], there is a strong correlation between chronic hepatitis B virus (HBV) infection and the occurrence of HCC. HBV X protein (HBx) is a well-known viral non-structural gene that operates as a multifunctional regulator by modulating activity of host cellular genes such as p53 [11], [12], [13] and transactivating some transcription factors including AP-1, NF-κB, CREB, and TBP [14], [15]. Moreover, HBx is involved in the activation of multiple signaling pathways linked to cell proliferation and survival, such as RAS/RAF/MAPK, MEKK1/JNK, and PI3K/Akt [16], [17], [18]. Chronic inflammation is one of the key conditions of persistent HBV infection and has been implicated in tumor development [19], [20], [21]. The proinflammatory cytokines and chemokines, such as tumor necrosis factor α (TNF-α), IL-1, IL-6, and IL-8, produced in microenvironments, have been known to promote tumor development [22], [23]. TNF-α is considered one of the most important factors involved in inflammation-mediated tumorigenesis [24], [25], [26], and the transcription factor NF-κB, a downstream signaling transducer of TNF-α, has been implicated in oncogenesis by promoting expression of genes related to cell proliferation and survival [27]. Activation of the inhibitor of nuclear factor κB (IκB) kinase (IKK) by TNF-α phosphorylates IκBs and promotes degradation of IκBs, resulting in nuclear translocation of NF-κB and induction of NF-κB downstream genes [28], [29]. The involvement of the IKK/NF-κB pathway in HBV-induced hepatitis and HCC is well documented [30], [31], [32], whereas effects of IKKs independent of NF-κB on tumorigenesis have also been found [33], [34], [35]. It was recently reported that IKKβ increased tumor development and tumor angiogenesis by activating the mTOR signaling pathway through inhibiting tuberous sclerosis 1 (TSC1) [36], [37], [38]. Moreover, aberrant activation of the mTOR/ribosomal protein S6 kinase 1 (S6K1) signaling pathway increased cell proliferation and angiogenesis in a rat HCC model [39], [40]. In the present study, we investigated whether HBx can modulate IKKβ to inactivate TSC1’s inhibition on mTOR so that it contributes to HCC development. We found that HBx modulated IKKβ/TSC1/mTOR signaling and up-regulated cell proliferation and VEGF production in both unstimulated and TNF-α-stmulated hepatoma cells. We further used an HBx transgenic mouse model to verify whether HBx upregulates IKKβ/TSC1/mTOR signaling *in vivo*, and to examine the association of upregulated IKKβ/TSC1/mTOR signaling with increased VEGF expression and angiogenesis in liver tumorigenesis. Furthermore, we investigated the status of IKKβ/TSC1/mTOR signaling in specimens from HBV-associated human hepatomas, and analyzed the relationship between the status of IKKβ/TSC1/mTOR signaling and the prognosis of HCC patients. We conclude that IKKβ activates mTOR signaling through TSC1 suppression to contribute to one crucial mechanism underlying HBx-dependent pathogenesis of HCC.

**Figure 1:**
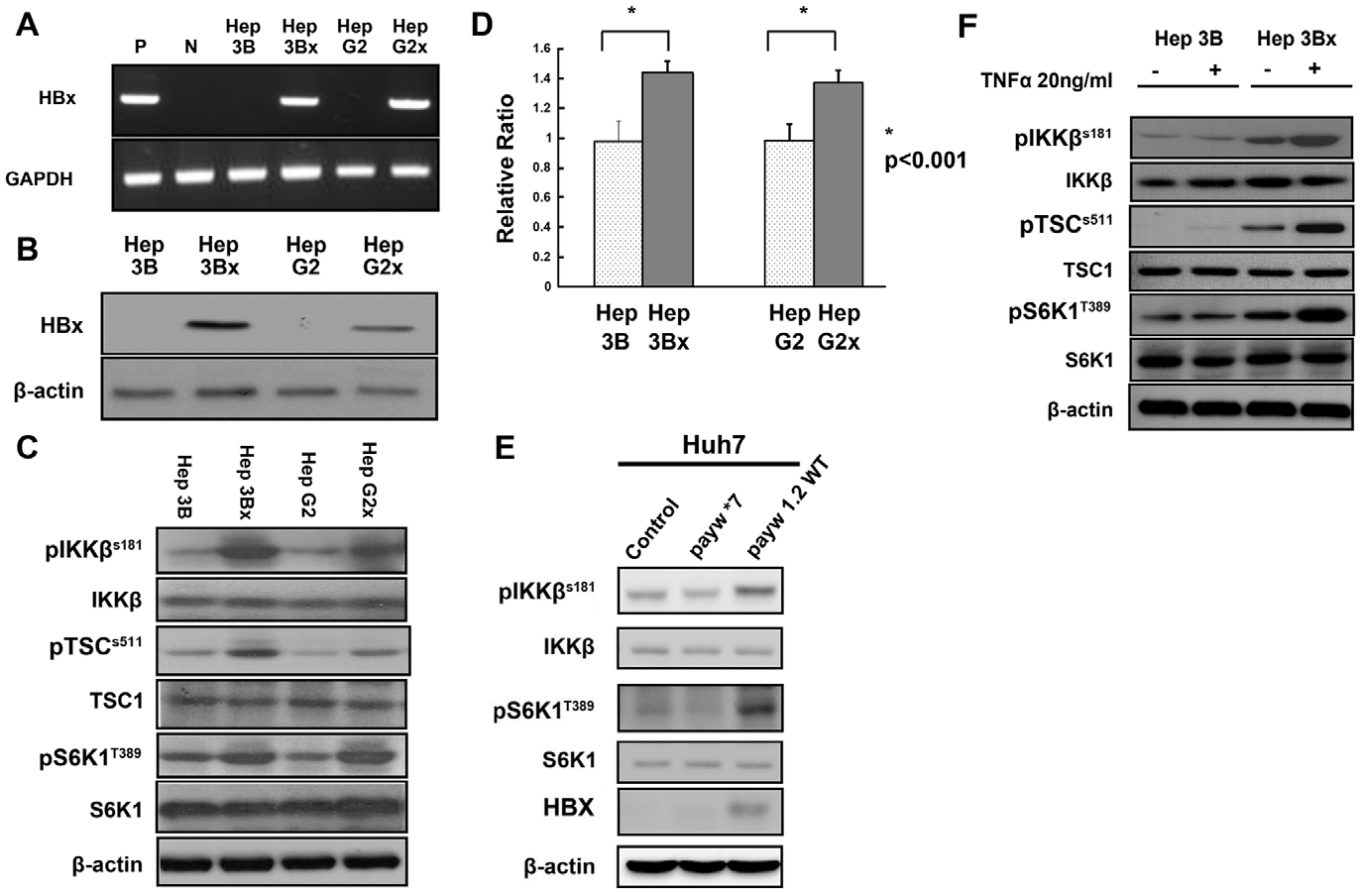
The IKKβ/TSC1/mTOR signaling pathway is activated by HBx. (A). Expression of HBx mRNA in Hep3Bx, HepG2x, and parental Hep3B and HepG2 cells was detected using semi-quantitative RT-PCR. Levels of GAPDH mRNA were used as an internal control. RNAs of a HBV-positive patient’s serum (P) and RNAs of a control HBV-negative serum (N) were used as controls. (B). Levels of HBx protein were detected in lysates of Hep3Bx, HepG2x, and parental Hep3B and HepG2 cells using Western blotting by antibody specific against HBx protein and β-actin. (C). Levels of pIKKβ(S181), pTSC1 (S511), pS6K1 (T389), total IKKβ, total TSC1, total S6K1, and β-actin were assessed in lysates of Hep3Bx, HepG2x, and parental Hep3B and HepG2 cells using Western blotting by specific antibody as indicated. (D). Data shown are ratios of viable cells in Hep3Bx and HepG2x cells relative to that in Hep3B and HepG2 cells (set as 1), respectively, at 24 h after seeding using MTT assay. (E). Levels of pIKKβ (S181), pS6K1 (T389), total IKKβ, total S6K1, HBx, and β-actin were assessed in lysates of Huh7 cells transfected with empty vector alone, payw1.2WT, or payw*7. (F). Levels of pIKKβ (S181), pTSC1 (S511), pS6K1 (T389), total IKKβ, total TSC1, total S6K1, and β-actin were assessed in lysates of Hep3B and Hep3Bx with or without TNF-α treatment using Western blotting as described earlier.

## Methods

### Plasmid and Cell Lines

The pcDNA6.0-HBx plasmid was constructed by cloning the cDNA product of the HBx gene into the pcDNA6.0 expression vector. The cDNA of the HBx gene was obtained by preparing RNA from serum of an HBV (+) patient followed by a reverse transcription-polymerase chain reaction (RT-PCR) using reverse transcriptase (SuperScript III; Invitrogen), oligo(dT) primers, and HBx primers: Forward 5′–AAGCTTGCTGCTCGGGTGTGCTGCCAA–3′ and Reverse 5′–GGTACCGG CAGAGGTGAAAAAGTTGCA–3′. The sequence of the HBx cDNA was confirmed by sequencing analysis. The expression vectors payw1.2WT and payw*7 [41] for wildtype and HBx-defective HBV genome, respectively, were kind gifts from Dr. Jack R. Wands (The Warren Alpert Medical School of Brown University).

**Figure 2:**
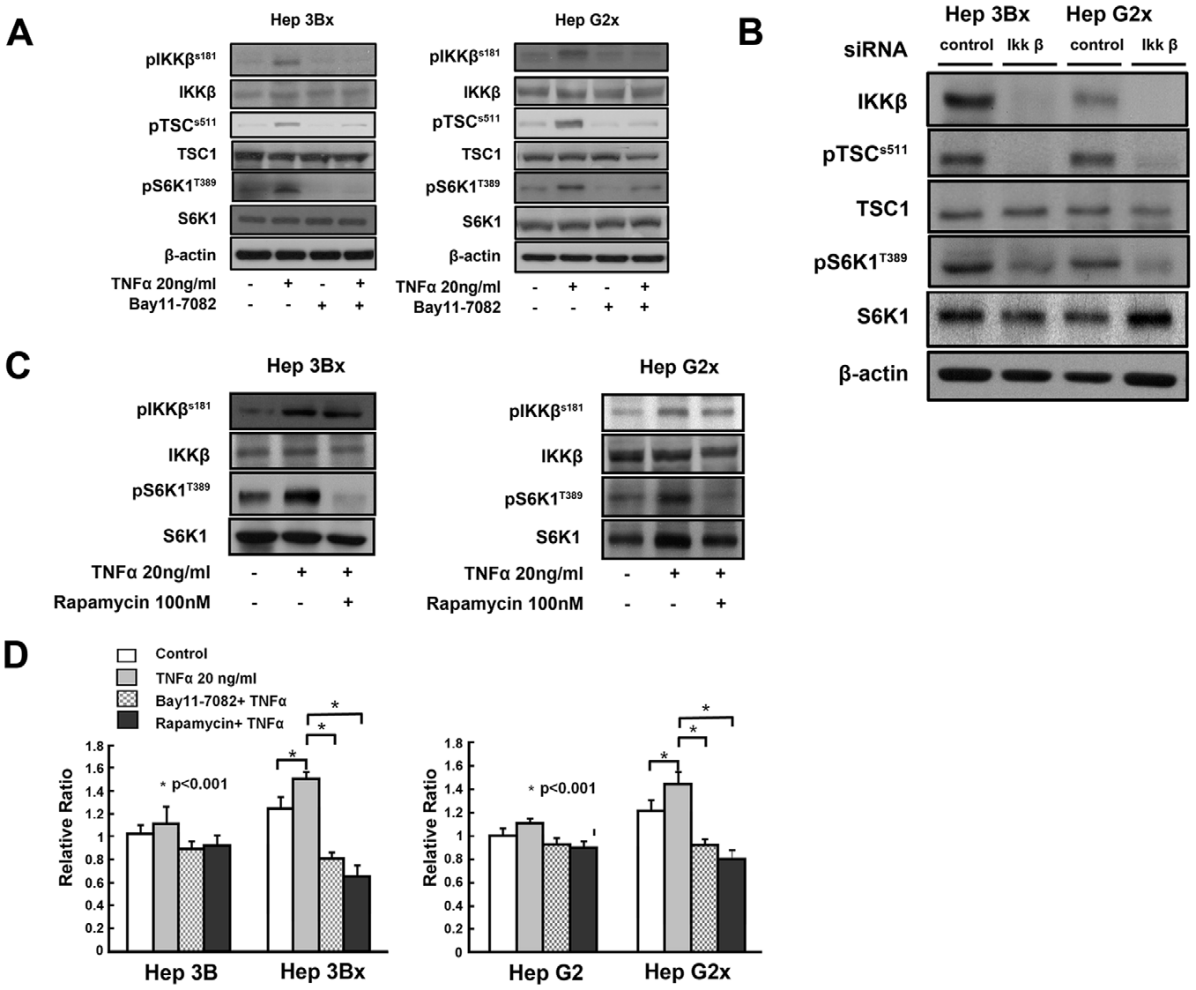
TNF-α-stimulated increases of pTSC1 (S511), pS6K1 (T389) and cell proliferation in Hep3Bx and HepG2x cells are blocked by the IKKβ inhibitor Bay 11-7082, siRNA specific for IKKβ, and the mTOR inhibitor rapamycin. (A). Lysates of Hep3Bx and HepG2x cells treated with or without TNF-α in the presence or absence of Bay 11-7082 were analyzed for levels of pIKKβ (S181), pTSC1 (S511), pS6K1 (T389), total IKKβ, total TSC1, total S6K1, and β-actin using Western blotting as described earlier. (B). Lysates of Hep3Bx and HepG2x cells with transfection of IKKβsiRNAs or control siRNAs were assessed for levels of pTSC1 (S511), pS6K1 (T389), total IKKβ, total TSC1, total S6K1, and β-actin. (C). Lysates of Hep3Bx and HepG2x cells treated with or without TNF-α in the presence or absence of rapamycin were analyzed for levels of pIKKβ (S181), pS6K1 (T389), total IKKβ, and total S6K1. (D). Data shown are ratios of viable cells in Hep3B, Hep3Bx, HepG2, and HepG2x cells treated with or without TNF-α in the presence or absence of Bay11-7082 or rapamycin relative to that in Hep3B and HepG2 cells without any treatment (set as 1), at 24 h after seeding using MTT assay. Data are shown as means ± S.D. of three experiments. Comparisons were made between different groups as indicated. *P<0.001 is determined by X test.

Hep3Bx and HepG2x cells were derivatives of human hepatoma Hep 3B and Hep G2 cells (both were from ATCC), respectively, stably expressing the HBx gene, and were established by transfecting Hep3B and HepG2 cells with pcDNA6.0-HBx using Lipofectamine (Invitrogen) followed by drug selection [42]. These cells were maintained at 37°C in a 5% CO_2_ incubator with Dulbecco’s modified Eagle’s/F12 medium plus 10% fetal bovine serum.

### Antibodies and Western Blotting

The primary antibodies used in this study were anti-TSC1 (37-0400; Zymed Laboratories, Inc., San Francisco, CA), anti-phosphorylated S6 kinase (T389) (9205; Cell Signaling Technology, Inc., Beverly, MA), anti-S6 kinase (2215; Cell Signaling), anti-phosphorylated IKKβ (S181) (2681; Cell Signaling), anti-IKKβ (2684; Cell Signaling), anti-HBx (ab235; Abcam Co., Cambridge, UK), anti-VEGF-A (Santa Cruz Biotechnology, Inc., Santa Cruz, CA), anti-CD31 (ab28364; Abcam Co., Cambridge, UK), and anti-actin (A2066; Sigma-Aldrich Co., St Louis, MO). The rabbit polyclonal antibody against the phospho-S511 of TSC1 was a kind gift from Dr Mien-Chie Hung (MD Anderson Cancer Center, Houston, TX). The expression of IKKβ, pIKKβ (S181), TSC1, pTSC1 (S511), S6K1 or pS6K1 (T389) was detected in cell lysates prepared from cells lysed as described previously [37]. Fifty micrograms of total protein lysates were resolved using sodium dodecyl sulfate polyacrylamide gel electrophoresis (SDS-PAGE), transferred to polyvinylidene difluoride (PVDF) membranes, and then probed with specific antibodies and HRP-conjugated secondary antibodies. Immunoblots were then developed using enhanced chemiluminescence.

**Figure 3:**
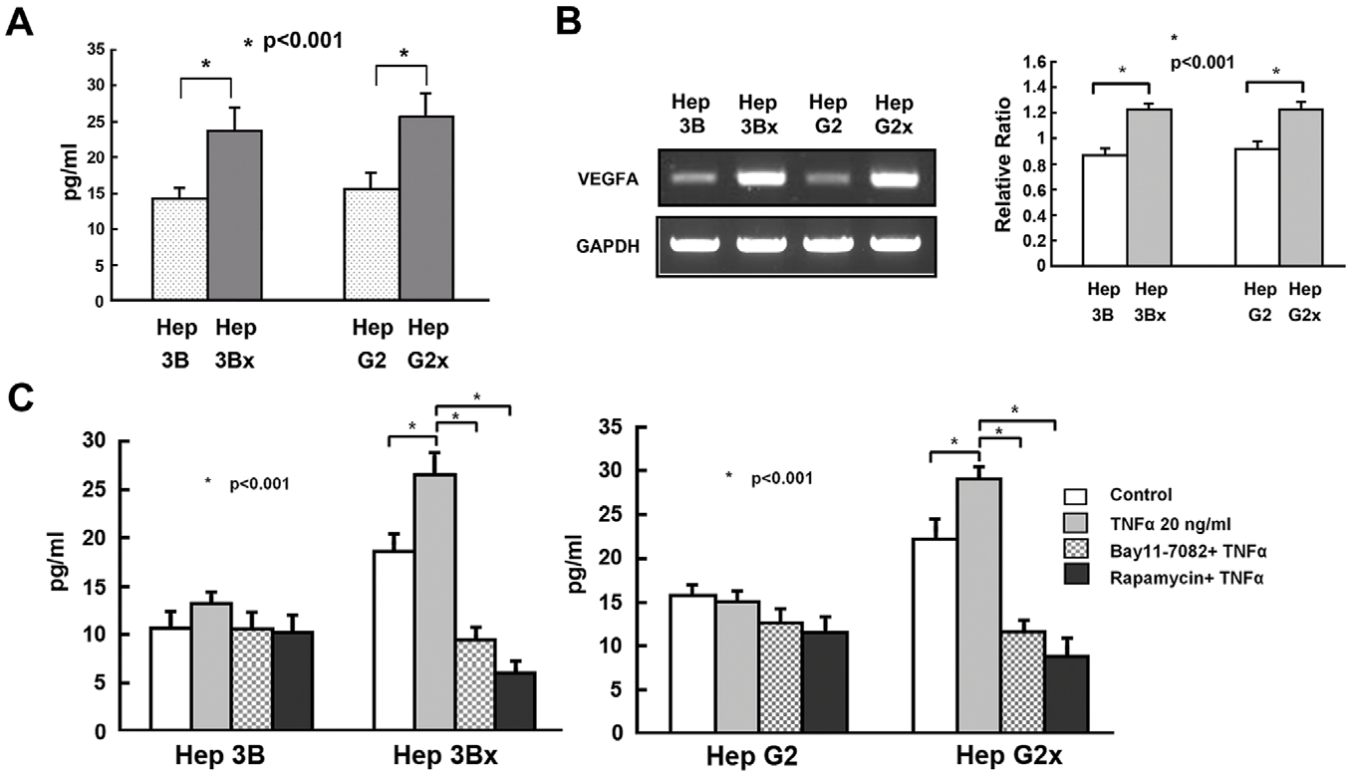
Expression of VEGF is increased in Hep3Bx and HepG2x cells and is further enhanced by TNF-α and blocked by IKKβ inhibitor Bay 11-7082 or the mTOR inhibitor rapamycin. (A). The expression levels of secreted VEGF in the culture medium of Hep3B, Hep3Bx, HepG2, and HepG2x cells were measured by ELISA assay as described in the Methods. (B). The expression levels of VEGFA mRNA were assessed in Hep3B, Hep3Bx, HepG2, and HepG2x cells using semi-quantitative RT-PCR (left) or real-time RT-PCR (right) as described in the Methods. (C). The amounts of secreted VEGF in the culture medium of Hep3B, Hep3Bx, HepG2, or HepG2x cells treated with or without TNF-α in the presence or absence of Bay11-7082 or rapamycin were measured by ELISA assay. Data are shown as means ± S.D. of three experiments. Comparisons were made between different groups as indicated. *P<0.001 is determined by X test.

### Inhibitor Treatment and Knockdown of Gene Expression by siRNAs

BAY 11-7082 and rapamycin were purchased from Calbiochem (San Diego, CA), and recombinant human TNF-α was from Roche Applied Sciences (Indianapolis, IN). Hep3B, Hep3Bx, HepG2, and HepG2x cells were grown in complete medium and exposed to 20 µg/ml TNF-α (Roche, Indianapolis, IN) with or without pretreatment of 40 µM BAY 11-7082 for 45 min (Calbiochem, San Diego, CA) or 100 nM Rapamycin (Calbiochem, San Diego, CA) for 3 h.

The MTT assay was used to measure cell proliferation and viability in 5,000 cells seeded onto 96-well plates, treated with TNF-α with or without BAY 11-7082 or Rapamycin for 24 h. The amount of VEGF protein in the culture supernatants was assessed using ELISA (BioSource; Invitrogen Corp., Carlsbad, CA) according to the manufacturer’s instructions. IKKβ siRNAs (Smartpool [M-003503]; Upstate Biotechnology, Charlottesville, VA) and control siRNAs (Smartpool [D-001206-13-05]; Upstate) were transfected into cells using Lipofectamine to knock down IKKβ expression.

**Figure 4:**
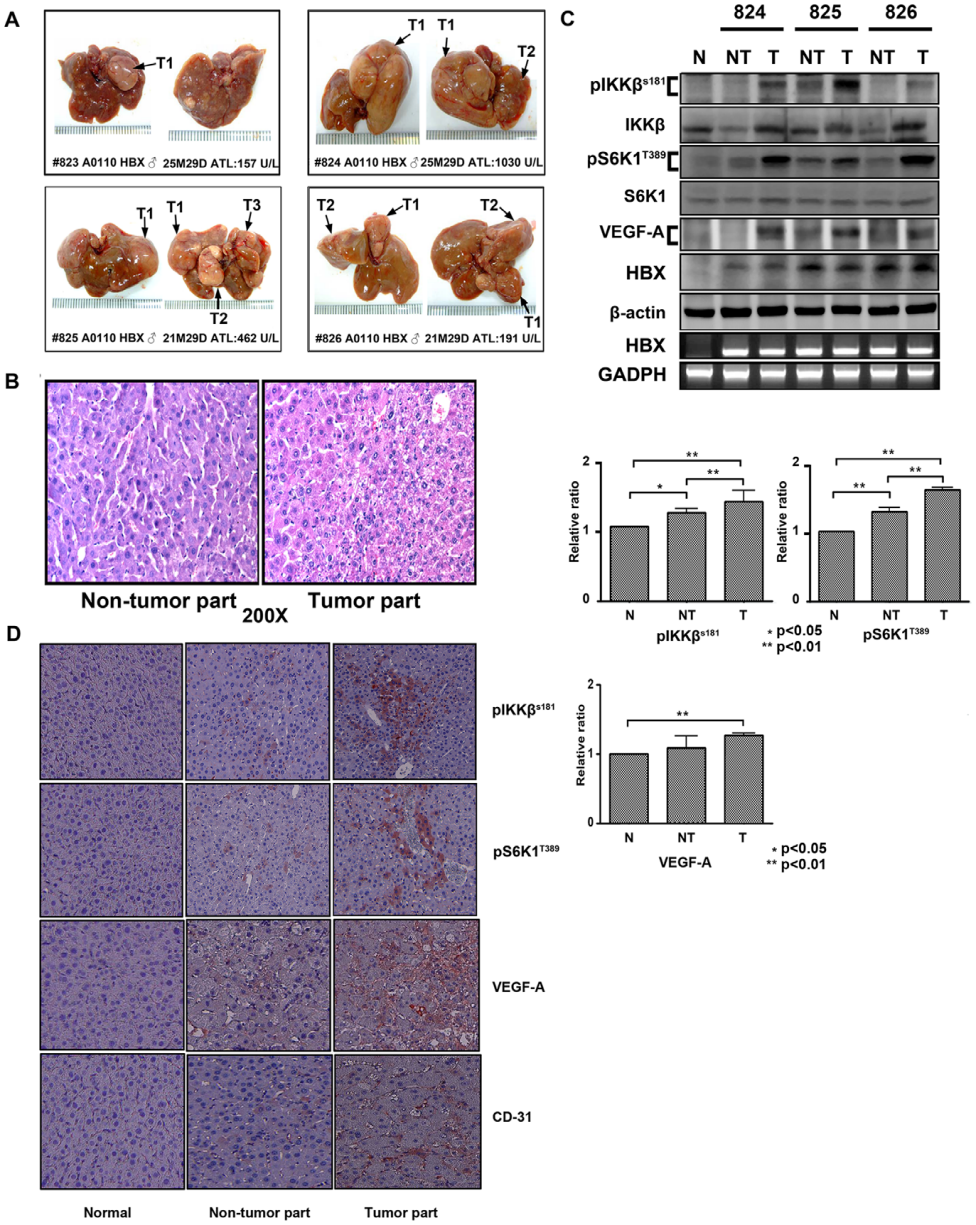
Increased expression levels of pIKKβ(S181), pS6K1(T389), and VEGF-A in liver tissues of HBx transgenic mice. (A). A gross view of representative liver tumors (T1, T2, T3) developed in HBx transgenic mice in several months of breeding. The ALT values are shown. (B). The H&E staining of non-tumor and tumor parts in HBx transgenic mice. (C). The expression levels of pIKKβ (S181), IKKβ, pS6K1 (T389), S6K1, VEGF-A, HBx, and β-actin detected by Western blotting in non-tumor and tumor parts of liver tissues of three HBx transgenic mice (#824, #825, and #826) were compared to the normal liver tissues of the wild-type age-matched mouse. The HBx mRNA levels were also measured by RT-PCR and the GAPDH mRNA levels were used as an internal control. The relative levels of pIKKβ (S181), pS6K1 (T389), and VEGF-A were quantified by densitometry and normalized with total IKKβ, total S6K1, and actin. Results are shown as ratios of average levels of pIKKβ (S181) pS6K1 (T389), and VEGF-A in non-tumor and tumor parts of liver tissues of three HBx transgenic mice (#824, #825, and #826) relative to that in the normal liver tissues of the wild-type age-matched mouse (set as 1). Data are shown as means ± S.D. of measurements of three mice. (D). Immunohistochemistry analyses show expression levels of pIKKβ (S181), pS6K1 (T389), VEGF-A, and CD31 in normal liver tissues of the wild-type mouse, and non-tumor and tumor parts of liver tissues of HBx transgenic mice. One representative data are shown. N = 3.

### Real-time RT-PCR and Quantitative Real-time RT-PCR for mRNAs

Total RNA was extracted from cells 48 h after transfection using Rezol (ProTech Technology Enterprise Co., Ltd., Taipei, Taiwan) according to the manufacturer’s instructions. Subsequently, 4 µg RNA was converted to cDNA using SuperScript III, oligo (dT) primers, and PCR using the following primers: HBx Forward 5′–AAGCTTGCTGCTCGGGTGTGCTGCCAA–3′ and Reverse 5′–GGTACCGG CAGAGGTGAAAAAGTTGCA–3′; VEGFA Forward 5′–CATGAACTTTCTGC TGTCTTGG–3′ and Reverse 5′–CATTTGTTGTGCTGTAGGAAGC–3′; GAPDH Forward 5′–TGAAGGTCGGAGTCAACGGATTTGGT–3′ and Reverse 5′–CATG TGGGCCATGAGGTCCACCAC–3′. For real time RT-PCR reactions, 1 µg of total RNA from each sample were used in the RT reaction (M-MLV Reverse Transcriptase; Invitrogen). The TaqMan gene expression real time PCR assays (ABI PRISM 7900 HT Sequence Detection System; Applied Biosystems) were used to assess the mRNA expression levels of the endogenous VEGFA and GAPDH (Applied Biosystems, Foster City, CA, USA; assay ID: Hs00900055_m1 for VEGFA and Hs99999905_m1 for GAPDH). Expression analysis was done in triplicate for each sample. In each run, the endogenous control gene (GAPDH) and one no-template-control (NTC) were also run in triplicate. The fold difference for each sample was obtained using the following equation: 2**^–ddCt^**. Ct is the threshold cycle.

### HBx Transgenic Mice, Tissue Preparation, and Immunohistochemical Analysis

The lines of HBx transgenic mice used in this study were established and described elsewhere [43]. The HBx transgenic mice were bred in a specific pathogen-free environment and all mouse experiments complied with the guidelines in the “Guide for the Care and Use of Laboratory Animals” (NIH publications 86-23 revised 1985) and were approved by the Institutional Animal Care and Use Committee (IACUC) of College of Medicine, National Cheng Kung University (Approval Number: 98129). The HBx transgenic mice developed hepatic tumor after 13 to 16 months of age. To ensure the mice could develop hepatic tumor, the transgenic mice were bred for up to 21 months, and approximately 90% incidence of HCC was observed in HBx transgenic male mice at an age of 19–20 months. After the mice had developed liver cancer, the mice were sacrificed, and liver tissue was collected, extracted, fixed, and stained with hematoxylin and eosin as previously described [42]. pIKKβ, pS6K1, VEGF-A, and CD31 expression was immunohistochemically detected on paraffin-embedded liver sections (3 µm) using specific antibodies mentioned earlier.

**Figure 5:**
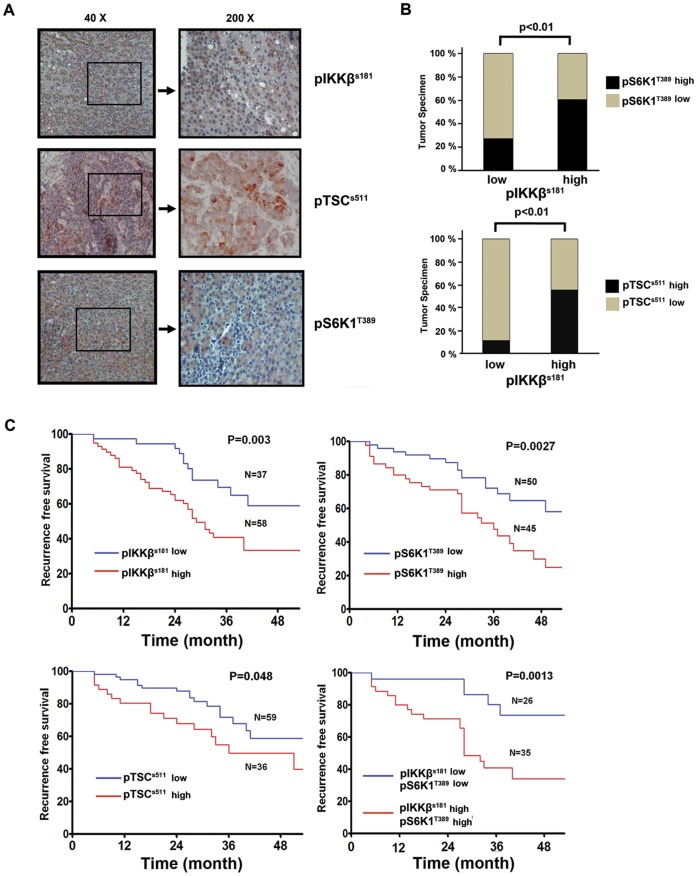
Positive association between pIKKβ(S181), pTSC1(S511), and pS6K1(T389) in HBV-associated human HCC specimens. (A). Immunohistochemistry analysis of pIKKβ (S181), pTSC1(S511) and pS6K1(T389) in tumor tissues of 95 human HBV-associated HCC specimens. Results of one representative specimens stained by specific antibodies are shown. (B). Upper graph shows percentages of specimens with low or high pIKKβ (S181) expression in which pS6K1 (T389) expression was high or was not observed (low). Lower graph shows percentages of specimens with low or high pIKKβ (S181) expression in which pTSC1 (S511) expression was high or was not observed (low). Positive correlations was noted between pIKKβ (S181) and pS6K1 (T389) (*P<0.01) and between pIKKβ (S181) and pTSC1 (S511) (*P<0.01)**.** (C) The Kaplan-Meier disease-free survival curves show that expression of pIKKβ (S181) (*p* = 0.003), pTSC1 (S511) (*p* = 0.048), or pS6K1 (T389) (*p* = 0.0027) is associated with early tumor recurrence. Co-expression of pIKKβ (S181) and pS6K1 (T389) (*p* = 0.0013) was a better predictor of patients’ recurrence-free time survival in HCC patients who received curative surgery for up to 48-month investigation.

### Patients’ Characteristics

Institutional Review Board of the Human Investigation Committee of College of Medicine, National Cheng Kung University approved the study. Written informed consent was obtained from patients participating in this study. A total of ninety-five patients admitted to National Cheng-Kung University Hospital (Tainan, Taiwan) with HBV-associated HCC who received curative surgery between 1 January 2003 and 31 December 2006 were enrolled, and samples of their resected liver tumor tissue were assembled in a tissue microarray. All 95 patients showed positive for serum HBV surface antigen and for HBx in tumor specimens analyzed using PCR, and negative for antibodies to the hepatitis C virus. The patients were regularly followed up at clinic visit every 1 to 3 months after curative surgery. The patients included 70 (73.7%) males and 25 (26.3%) females with age range of 44 to 77 years (mean age 60.7 years). The median follow-up time was 38 months (range, 6 to 53 months). At the end of the follow-up, 28 patients had died of disease. The 1-year disease specific survival rate was 92.1% and 3-year disease specific survival rate was 74.2%.

### Immunohistochemistry and Clinical Association Study

Immunohistochemical staining for pIKKβ, pTSC1, and pS6K1 protein expression was examined on adjacent 4-µm formalin-fixed paraffin-embedded tissue sections. An experienced gastrointestinal pathologist reviewed all specimens. Amino-ethylcarbazole chromogen was used for visualization. The positive protein staining was only considered in cytoplasmic immunoreactivity of cancer cells on a semi-quantitative scale that combined staining intensity and percentage of positively stained cells. Staining intensity was evaluated as low (0–10% positive cancer cells) or high (>10% positive cancer cells). Statistical analyses were done using a χ^2^ test, Fisher’s exact test, or Kaplan-Meier survival test. Significance was set at P<0.05.

## Results

### HBx Upregulates IKKβ and mTOR Activity

We established human hepatoma Hep 3Bx and Hep G2x cell lines which stably express HBx to investigate the effects of HBx overexpression on IKKβ/TSC1/mTOR signaling. The expression of HBx in Hep3Bx and Hep G2x was confirmed by both RT-PCR and Western blotting analysis (Fig. 1A, B). Next, we evaluated whether HBx overexpression affected levels of phospho-IKKβ (pIKKβ) (S181), phospho-TSC1 (pTSC1) (S511), and mTOR downstream phospho-S6K1 (pS6K1) (T389). While there were no significant differences in total protein levels of IKKβ, TSC1, S6K1 in cells with or without overexpression of HBx (Fig. 1C), we found overexpression HBx enhanced levels of pIKKβ, pTSC1, and pS6K1. Consistently, we observed significantly increased proliferation in cells stably expressing HBx compared with control cells expressing vector only (Fig. 1D). To verify the role of HBx in enhancing levels of pIKKβ and pS6K1 in a more natural setting mimicking HBV infection, we transfected Huh7 cells with empty vector alone, wildtype HBV full genome expression vector payw1.2WT, or payw*7 which is a paywWT mutant vector harboring an orchre termination signal after codon 7 in the HBx open reading frame [41]. We found that levels of phosphorylation of IKKβ and S6K1 in cells transfected with wildtype HBV expression vector payw1.2WT were higher than that in cells transfected with HBx defective mutant payw*7 or vector only (Fig. 1E). Moreover, when cells were treated with the proinflmmatory cytokine TNF-α, overexpression of HBx synergistically enhanced TNF-α-stimulated pIKKβ, pTSC1, and pS6K1 (Fig. 1F). These results demonstrate that HBx upregulates basal and TNF-α-induced IKKβ and mTOR activity.

**Table 1:** Recurrence free survival in multivariate analysis.

Var	Hazard Ratio(95% CI)	P-value
Gender		
Male	1.0	
Female	0.5	0.08
	(0.3–1.1)	
Age		
<50 years	1.0	
≧50 years	0.7	0.41
	(0.4–1.5)	
Albumin		
Albumin <3.5 g/dl	1.0	
Albumin ≧3.5 g/dl	0.6	0.17
	(0.3–1.2)	
AFP		
AFP<400 ng/ml	1.0	
AFP>400 ng/ml	0.5	0.08
	(0.2–1.1)	
Differentiation		
Well	1.0	
Moderate	0.9	0.77
	(0.5–1.7)	
Poor	1.3	0.55
	(0.6–3.1)	
Primary tumor T stage		
T1	1.0	
T2& T3&T4	1.2	0.59
	(0.6–2.4)	
BCLC		
A stage	1.0	
B stage	1.8	0.07
	(1.0–3.2)	
pIKKβ(S181)		
Low expression	1.0	
High expression	2.37	0.003
	(1.33–4.2)	
pTSC1(S511)		
Low expression	1.0	
High expression	2.07	0.048
	(1.01–4.26)	
pS6K1(T389)		
Low expression	1.0	
High expression	2.56	0.0027
	(1.39–4.74)	
pIKKβ(S181) & pS6K1(T389)		
Low expression & Low expression	1.0	
High expression & High expression	4.11	0.0013
	(1.65–7.92)	

### HBx Deregulates TSC1/mTOR Signaling and Increases Cell Proliferation through IKKβ

We found that HBx enhanced basal and TNF-α-stimulated IKKβ and mTOR activity, and concomitantly increased phosphorylation and inactivation of TSC1 (Fig. 1). We hypothesized that the effect of HBx on mTOR was mediated by IKKβ-mediated phosphorylation and inactivation of TSC1. The Hep3Bx and HepG2x cells were treated with the IKKβ inhibitor Bay11-7082 in the presence or absence of TNF-α, and the levels of pIKKβ (S181), pTSC1 (S511), and pS6K1 (T389) were examined. In agreement with our hypothesis, inhibition of IKKβ by Bay11-7082 abolished the TNF-α-stimulated phosphorylation of TSC1 and S6K1 in both Hep3Bx and HepG2x cells (Fig. 2A), the finding of HBx-upregulated mTOR activity mediated by IKKβ was in parallel confirmed using a specific IKKβ siRNA to knockdown IKKβ expression (Fig. 2B). To confirm that the HBx-mediated increase in phosphorylation of S6K1 was through mTOR activation, cells were treated with mTOR inhibitor rapamycin when stimulated with TNF-α. As shown in Fig. 2C, basal and TNF-α-stimulated phosphorylation of S6K1 was abolished by rapamycin treatment in both Hep3Bx and HepG2x cells. Consistently, TNF-α-stimulated cell proliferation in Hep3Bx and HepG2x cells was completely blocked by treatment with Bay11-7082 or rapamycin (Fig. 2D). These results suggest that HBx activates mTOR activity and cell proliferation through IKKβ-mediated inactivation of TSC1.

### HBx Increases VEGF Production through IKKβ/TSC1/mTOR Signaling

Since activation of mTOR pathway could up-regulate the angiogenesis process [36], [37], we used IKKβ inhibitor Bay 11-7082 and mTOR inhibitor rapamycin to clarify whether HBx could induce VEGF production through IKKβ/TSC1/mTOR signaling. The expression level of VEGF in cell culture supernatant was checked by ELISA, and we observed increased VEGF production in the Hep 3Bx and Hep G2x cells compared with control Hep 3B and Hep G2 cells (p<0.001) (Fig. 3A). Results of semi-quantitative and real-time RT-PCR assay showed that the expression of VEGF-A messenger RNA was also upregulated in Hep 3Bx and Hep G2x cancer cells (Fig. 3B). In order to further clarify whether HBx enhanced VEGF production was related to IKKβ/TSC1/mTOR signaling, effects of TNF-α (20 ng/ml) combined with or without pretreatment of IKKβ inhibitor Bay 11-7082 or mTOR inhibitor rapamycin were examined. We found that TNF-α-enhanced VEGF production in Hep 3Bx and Hep G2x was substantially suppressed by treatment with Bay 11-7082 or rapamycin, as compared with that in control Hep 3B and Hep G2 cells (p<0.001) (Fig. 3C). These observations indicate that HBx increases the VEGF production of the hepatoma cells due to modulation of the IKKβ/TSC1/mTOR pathway.

### HBx-mediated Upregulation of IKKβ/TSC1/mTOR Signaling is Associated with Liver Tumorigenesis in HBx Transgenic Mice

To further understand the contribution of the HBx-modulated IKKβ/TSC1/mTOR signaling pathway in liver tumorigenesis, we used an HBx transgenic mouse model which has been shown to develop liver tumors [42] (Fig. 4A, B). We found that the average expression levels of both pIKKβ and pS6K1 were higher in both the non-tumor parts and liver tumor tissues of the HBx transgenic mice liver compared with the normal liver tissues of the wild-type mice (Fig. 4C). Concurrently, significantly higher levels of VEGF-A were also found in liver tumor tissues of HBx transgenic mice compared with the normal liver tissues of the wild-type mice (Fig. 4C). In agreement with the results of Western blottings, immunohistochemical staining revealed higher expression levels of pIKKβ, pS6K1, VEGF-A, and CD31 in liver tumors of HBx transgenic mice compared with adjacent non-tumor parts and wild-type mice liver tissues (Fig. 4D). Thus, findings of the HBx transgenic mouse model support that HBx enhances the IKKβ/mTOR signaling pathway and promotes VEGF–A production and new vessels formation.

### Immunohistochemical Staining Reveals Positive Correlations between pIKKβ and pTSC1, and between pIKKβ and pS6K1 in HBV-associated Human HCC Tissue Specimens

To validate the relevance of the upregulation of TSC1/mTOR pathway via IKKβ signaling in HBV-associated human HCC, we evaluated the expression of pIKKβ, pTSC1, and pS6K1 in a tissue microarray of 95 HBV-associated human HCC tissue specimens by immunohistochemical staining. All 95 patients who received curative surgery were positive for both serum HBV surface antigen and HBx, but negative for antibodies to hepatitis C virus (data not shown). Expression of pIKKβ, pTSC1, and pS6K1 was detected in human HCC tissue specimens (Fig. 5A). Analysis of immunohistochemical staining of 95 HCC tissue specimens revealed that pS6K1 was detected in 35 (60%) of the 58 specimens with high pIKKβ expression, but it was detected in only 10 (27%) of the 37 specimens with low pIKKβ expression, indicating that pS6K1 expression was positively associated with pIKKβ expression (P<0.01) (Fig. 5B, upper). More, pTSC1 expression was detected in 32 (55%) of the 58 specimens with high pIKKβ expression, but it was detected in only 4 (11%) of the 37 specimens with low pIKKβ expression (P<0.01) (Fig. 5B, lower). We next analyzed the correlation of the expression of pIKKβ (S181), pTSC1 (S511), and pS6K1 (T389) in HCC specimens with patients’ recurrence-free survival data. The Kaplan-Meier recurrence-free survival curves showed that high levels of pIKKβ (S181), pTSC1 (S511) and pS6K1 (T389) were associated with early recurrence of HCC (Fig. 5C). Multivariate analysis indicated that expression of pIKKβ (S181) (HR, 2.37; 95% CI, 1.33–4.2 [P = 0.003]), pTSC1 (S511) (HR, 2.07; 95% CI, 1.01–4.26 [P = 0.048]) and pS6K1 (T389) (HR, 2.56; 95% CI, 1.39–4.74 [P = 0.0027]) were predictors of patients’ recurrence-free time (Table 1). In addition, concomitant expression of pIKKβ (S181) and pS6K1 (T389) was a better predictor of patients’ recurrence-free survival (HR, 4.11; 95% CI, 1.65–7.92 [P = 0.0013]) than each factor alone (Table 1). Taken together, these data suggest that HBx-modulated IKKβ/TSC1/mTOR pathway may play a crucial role in HBV-associated human HCC development and progression.

## Discussion

The link between HBV and the development of hepatocellular carcinoma has been well established, but the pathogenic mechanism responsible for the transformation of normal hepatocytes to HCC is still far from understood, especially in view of the contribution of HBx to cancer progression. We have provided evidence that HBx activates IKKβ, which leads to inactivation of TSC1 and activation of mTOR/S6K1 and to the production of angiogenesis factor VEGF-A in HBx expressing hepatoma cells and liver tissues of HBx transgenic mice. The HBx-associated IKKβ/TSC1/mTOR signaling pathway may play a molecular switch that allows HBV-related HCC tumor progression. This is likely to be clinically relevant to pathogenesis as we found a statistically significant correlation between the phosphorylation of IKKβ and phosphorylation of TSC1, and between the phosphorylation of IKKβ and phosphorylation of S6K1 in HBV-associated HCC specimens.

Chronic hepatitis B virus (HBV) infection causes an inflammation process in the normal liver tissue, resulting in liver damage that may subsequently evolve into liver cirrhosis and tumor development. The proinflammatory cytokine TNF-α has been shown to play a promoting role for tumor development [21], [23], [44], and activation of NF-κB, a downstream signaling transducer of TNF-α, has long been implicated in the development of HBV-associated HCC [45], [46]. HBx has been shown to activate NF-κB by directly interacting with NF-κB [47], [48], up-regulating TNF-α expression [48], or promoting phosphorylation and degradation of IκB [50]. HBx was also shown to activate NF-κB and up-regulate genes involved in cell invasion by IKKβ activation [51]. Recently, HBx was shown to activate NF-κB through modulating TRAF2/TAK1 signaling cascade [52]. The mechanism by which HBx activates IKKβ remains unclear, but it is conceivable that HBx may activate IKKβ by up-regulating TNF-α receptor stimulation through increasing TNF-α expression [49] and/or by modulating the TRAF2/TAK1 signaling module to increase IKKβ activation [52]. Additionally, HBx may activate IKKβ through activation of Ras/Raf/MEK [53] or protein kinase C (PKC) pathway [54], which has been known to be associated with IKK activation. Nevertheless, HBx may use alternative mechanisms to activate IKKβ. IKKs’ activities independent of NF-κB have been reported [33], [34], [35], and IKKβ was found to phosphorylate TSC1 and block the inhibitory effect of TSC1 on mTOR activity, resulting in increased tumorigenesis and angiogenesis [36], [37], [38]. Consistent with these findings, our results demonstrate that HBx up-regulates IKKβ to deregulate TSC1/mTOR signaling and to promote cell proliferation and VEGF production. Moreover, activation of the mTOR signaling pathway has been found in a significant portion of HCC examined, and mTOR inhibition showed antitumoral effects, although the relationship between the status of HBx and mTOR activation was not known [39], [55], [56]. HBx was demonstrated to enhance VEGF expression by up-regulation of HIF-1α transcription [57], [58]. Our results showed that HBx can modulate IKKβ/TSC1/mTOR signaling to increase VEGF-A production and TNF-α further up-regulates VEGF-A production in Hep3B and HepG2 cells overexpressing HBx. Similarly, using an HBx transgenic mouse liver tumor model, we demonstrated higher expression of pIKKβ, pS6K1, and VEGF-A, and neovascularization in tumor tissues when compared with the non-neoplastic area of the HBx transgenic liver.

We demonstrated that treatment of liver cancer cells with mTOR inhibitor rapamycin and IKKβ inhibitor Bay11-7082 effectively blocked the HBx-induced cell proliferation and VEGF-A production. Currently available treatments for non-operable advanced HCC patients, including local ablation therapy (radiofrequency ablation and percutaneous ethanol injection), trans-arterial chemo-embolization (TACE), chemotherapy and radiotherapy, are ineffective, resulting in very poor survival rates of patients [2], [3]. Furthermore, our observations in specimens of the clinical HBV-associated HCC patients demonstrated that the expression of pIKKβ (S181), pTSC1 (S511), and pS6K1 (T389) in human HCC tissue samples was correlated with early tumor recurrence and poor patients’ survival. Moreover, we showed that the combination of pIKKβ (S181) and pS6K1 (T389) expression was a better predictor of survival. Our findings suggest that pIKKβ (S181) and pTSC1 (S511) and pS6K1 (T389) might be used as prediction of poor treatment outcomes of HCC patients. Therefore, HCC patients who have a significantly elevated risk of poor treatment outcomes should receive more intensive therapy; for example, surgery followed by adjuvant chemotherapy or target therapy.

In conclusion, we demonstrate that HBx deregulates TSC1/mTOR signaling through IKKβ and renders liver cancer cells more sensitive to TNF-α stimulation in activating mTOR downstream S6K1 activity through IKKβ signaling. Activation of the IKKβ/mTOR pathway occurs concomitantly with increased cell proliferation and angiogenesis, which may associate with the progression of the HCC. Consistently, blocking IKKβ or mTOR signaling with Bay 11-7082 or rapamycin, respectively, inhibits the liver cancer cell growth and VEGF-A production, suggesting that inhibitors of IKKβ or mTOR signaling may be useful as new therapeutics for the treatment of HBV-associated HCC.
